# Assessment of microbiological quality of food preparation process in some restaurants of Makkah city

**DOI:** 10.1016/j.sjbs.2021.06.050

**Published:** 2021-06-24

**Authors:** Mamdouh A. Bukhari, Talib M. Banasser, Mohammed El-Bali, Rasha A. Bulkhi, Razaz A. Qamash, Amal Trenganno, Maher Khayyat, Mohammed A. Kurdi, Ahmed Al Majrashi, Fayez Bahewareth

**Affiliations:** a Regional Laboratory of Makkah, Ministry of Health, Makkah, Saudi Arabia; b Faculty of Medicine, Umm-Al-Qura University, Makkah, Saudi Arabia; c King Faisal Hospital, Makkah, Ministry of Health, Makkah, Saudi Arabia

**Keywords:** Makkah restaurants, Microbiological quality, Food contact surfaces, Food poisoning bacteria

## Abstract

Microbiological contamination of food processing surfaces and utensils increases considerably the risk of food-borne illnesses via cross-contamination. Hence, the safety of served meals and beverages can be evaluated through the assessment of the microbiological quality of food contact surfaces in food-serving establishments. This study carried out in Makkah city aimed to assess the microbiological contamination levels on food processing surfaces and utensils in 43 restaurants from the 9 main districts in the city. A total of 294 swab preparations were taken from 16 types of food contact surfaces including cutting boards, food containers, knives, serving dishes and other utensils were examined. Ninety samples (31%) showed more than 10 CFU/cm^2^ which were considered positive for microbiological contamination. Meat chopping devices and cutting boards were found as the most contaminated food contact surfaces (60% and 50%), while washed serving dishes and fridge handles were the least contaminated (21% and 18%). Microorganisms detected in the study were *Klebsiella* spp. (18.7%), *Escherichia coli* (17,7%), *Staphylococcus aureus* (4,4%), *Pseudomonas* spp. (1.7%), *Proteus spp*. (0.7%), *Bacillus cereus* (0.7%), and *Candida* sp. (0.3%). *Klebsiella* spp. and *E. coli* were observed in at least one sample from each of the sixteen different food contact surfaces. The incidence of restaurants with contaminated food contact surfaces was significantly variable among the different districts, with a value as high as 57% in the most affected district and 20% in the less affected. No contamination with *Salmonella* spp. or *Listeria* spp. was detected, however, the detection of *Bacillus cereus*, a toxin-forming microorganism, in two different restaurants underlines the need for continuous microbiological assessment to ensure standard sanitation levels in restaurants and catering establishments of Makkah city.

## 1 Introduction

Food-borne illnesses are considered a major public health issue that causes an important burden of disease and mortality worldwide. Food-borne diseases also impact negatively the socioeconomic development and productivity of many sectors such as tourism and trading (Newman et al., 2015). In Saudi Arabia increasing in food poisoning cases reported especially in the Hajj season and summer months. Furthermore, in general, the food poisoning problem has become a very crucial subject internationally (Al-Mazrou, 2004). Control and prevention programs of food poisoning in the kingdom of Saudi Arabia facilities should focus on *Salmonella*, *E. coli* and *Bacillus cereus* as they are the most suspicious in most cases of food poisoning in the last decade (Al-Mazrous, 2004, Organji et al., 2015). In addition, many reports have documented the spread of highly resistant antibiotic bacteria in food and the environment such as *Staphylococcus aureus* and coagulase-negative *staphylococci* (Abulreesh and Organji, 2011, Dablool and Al-Ghamdi, 2011).

Food-borne illnesses are originated by consumption of foods and beverages contaminated by harmful physical, chemical, or biological agents. Generally, foodborne diseases are infectious, caused by many pathogenic species of bacteria, viruses, or parasites (Newell et al., 2010, Petruzzelli et al., 2010). Food-borne infections are majorly due to inadequate storage and processing of raw material, under-cooking, or bad hygienic habits. Although most infections are related to home-prepared food, food-borne outbreaks associated with food serving establishments and restaurants are a public health priority (Bhat et al., 2019). Numerous studies have reported that foods served by catering services were the source of several foodborne outbreaks (Osimani and Clementi, 2016). In 2018, 358 food poisoning outbreaks were reported in KSA of which 51% were due to food consumption from public sources such as restaurants and food-serving establishments and due in their majority to bacterial infections mainly *salmonella* (Saudi MOH, 2018). Foodborne disease outbreaks are important challenges facing health authorities, especially in highly visited places known for a massive demand on restaurants and catering establishments such as in Makkah city which annually visited by millions of pilgrims and where outbreaks of infectious diseases are of major concern (Shirah et al., 2017).

Microbiological contamination of foods served in restaurants can occur at any stage during storage, manipulation, and processing, or even at the serving stage. It can be originated by contaminated raw materials or cross-contamination from the air, water, dust, human and animal wastes, and many other sources (Osimani et al., 2013). Several studies reported that substandard practices in food handling and processing lead to food-borne illnesses and outbreaks (Garayoa et al., 2017, Lee et al., 2017). Thus, the assessment of hygiene standards appliance in food-processing and serving establishments becomes a mandatory tool for control and prevention of foodborne diseases. Microbiological assessment should rely, not only on food analysis but it is fundamental investigating all types of food-contact surfaces and utensils employed in food processing, preparation and serving. Food contact surface hygiene swabbing coupled with viable cell counting has been internationally used for such purposes. This method has proved to be efficient for the detection of contaminant microbes such as *Bacillus cereus, Candida* sp.*, E. coli, Klebsiella* spp.*, Proteus* spp.*, Pseudomonas* spp.*, Staphylococcus aureus,* and other bacteria species (Hawronskyj and Holah, 1997).

This study was carried out in Makkah city to assess the hygienic and sanitation level in several restaurants of variable categories randomly located in the nine main districts of the city. Assessment of microbiological quality onto sixteen types of food contact surfaces was carried out using the swabbing method, as one of the most efficient collection techniques for such investigations (Christison et al., 2008).

## 2 Material and methods

### 2.1 Study site

This study was carried out in Makkah city, one of the most visited cities in the world that receives millions of visitors coming for Hajj and Ummrah. Hundreds of restaurants and food serving establishments are located all around the city, in its different districts, to serve residents and visitors. This study was carried out to investigate 16 types of food contact surfaces in 43 randomly selected restaurants from 9 main districts in the city (Table 1).

**Table 1:** Distribution of examined food processing surface swabs collected from restaurants in different districts of Makkah Al-Mukarramah.

Serial	District code	Restaurant code (number of samples)	Number of samples/district
1	Aw.	Bro (8), mel (8), sub (8), bar (9), boc (9), sha (8), waq (9)	59
2	Bo.	Dom (6), qor (8)	14
3	Es.	Lit (4), ary (4), her (3), ray (5)	16
4	Fa.	zaa (8), qom (9), buk (9)	26
5	Hu.	Ara (9), buk (9), rok (10), boc (2)	30
6	Ka.	Ala (9), tai (9), dha (7)	25
7	Om.	Har (4), ara (10), bro (7), ken (7), bii (9)	37
8	Ro.	Her (4), ben (9), sha (4), bay (9), als (4), mak (5), fal (4), sub (4)	43
9	Sh.	Har (6), shz (7), twa (8), ray (8), far (5), bai (4), mcd (6)	44
Total	**9 districts**	**43 Restaurants**	**294**

### 2.2 Samples' collection

A total of 294 samples were collected by specialists in Makkah city municipality during January 2021. Samples were taken from 16 types of food contact surfaces used for storage, defrosting, cutting, chopping, baking, or other processing of meat, chicken, or vegetables to prepare ready-to-consume food and beverages. Types and numbers of sampled food contact surfaces are gathered in Table 2. Sampling was done by a swabbing method adapted from (Bell et al., 2005). Briefly, an area of 20 cm^2^ of food processing surfaces like cutting boards and the whole area of small utensils like knives, was swabbed using sterile swabs pre-moistened in buffered peptone water (BPW) during 10–15 sec by rotating the swabs in different directions. Two points were considered during sample collection; the suitable temperature of the surrounding environment where samples collected at 25 °C and the cleanliness of the restaurants which may influence the interpretation of the results. Collected swabs were properly labeled, immediately placed on an iced cool box to prevent bacterial growth, and transported on the same day to Makkah Regional Laboratory to be microbiologically investigated once samples were received.

**Table 2:** Types and numbers of examined food processing surfaces.

Serial	Type of food processing surface	N^0^ samples
1	Baking surface	13
2	Chicken container	6
3	Chicken cutting board	11
4	Chicken knife	9
5	Defrosting sink	24
6	Fridge handle	34
7	Meat chopping device	5
8	Meat cutting board	22
9	Meat knife	14
10	Multi-cutting board	17
11	Multi- knife	11
12	Serving dish	48
13	Vegetable chopping device	12
14	Vegetable's container	25
15	Vegetable cutting board	25
16	Vegetable's knife	18
Total	**16 types**	**294**

### 2.3 Identification and enumeration

Each collected swab was flooded in 1 ml BPW and thoroughly vortexed in aseptic conditions. Directly, 10 µl of the suspension was poured in duplicate into Blood agar and MacConkey Sorbitol agar Petri dishes, and into Selenite F-broth media which were sub-cultured after 6 h onto solid Xylose Lysine Deoxycholate Agar (XLD agar). All plates were incubated at 37 °C for 24 to 48 h. Enumeration of colonies was carried out using a colony counter, and colonies over 10 CFU/cm^2^ were considered as positive cultures as the Centers for Disease Control and Prevention (CDC) pointed that the interpretation of the environmental surface samples is self-assessment (Guidelines for Environmental Infection Control in Health-Care Facilities, 2015). The identification of bacteria and fungus was done by using Vitek 2 compact.

### 2.4 Statistical analysis

Collected data was plotted on excel sheets to display the results and graphs. Statistics analysis was performed using SPSS software.

## 3 Results

**Fig. 1:**
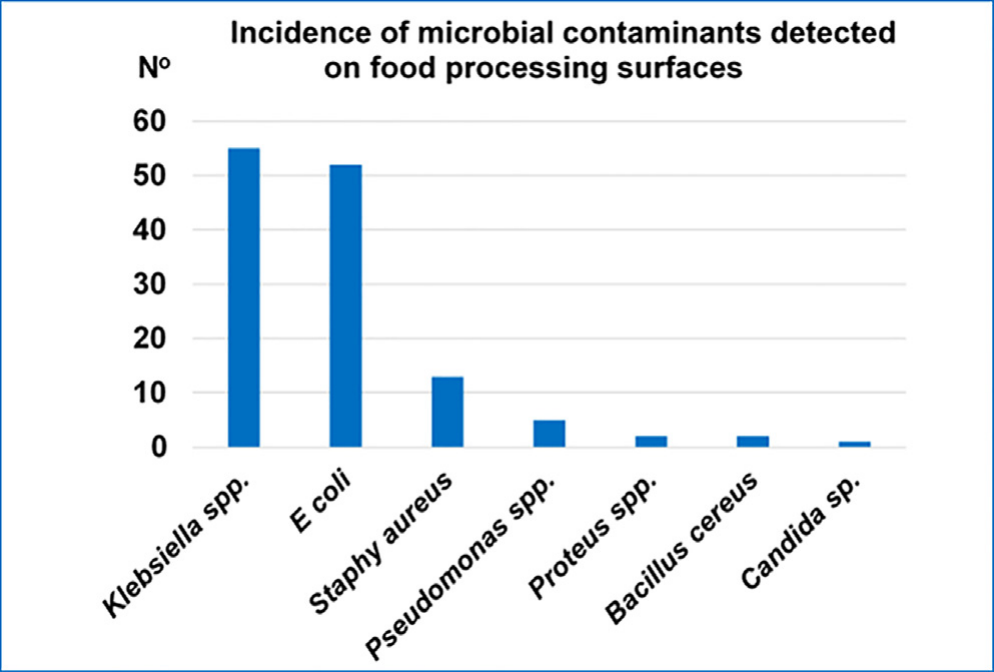
Incidence of contaminant microorganisms detected onto the 294 examined food contact surfaces from 43 restaurants in Makkah holy city.

**Fig. 2:**
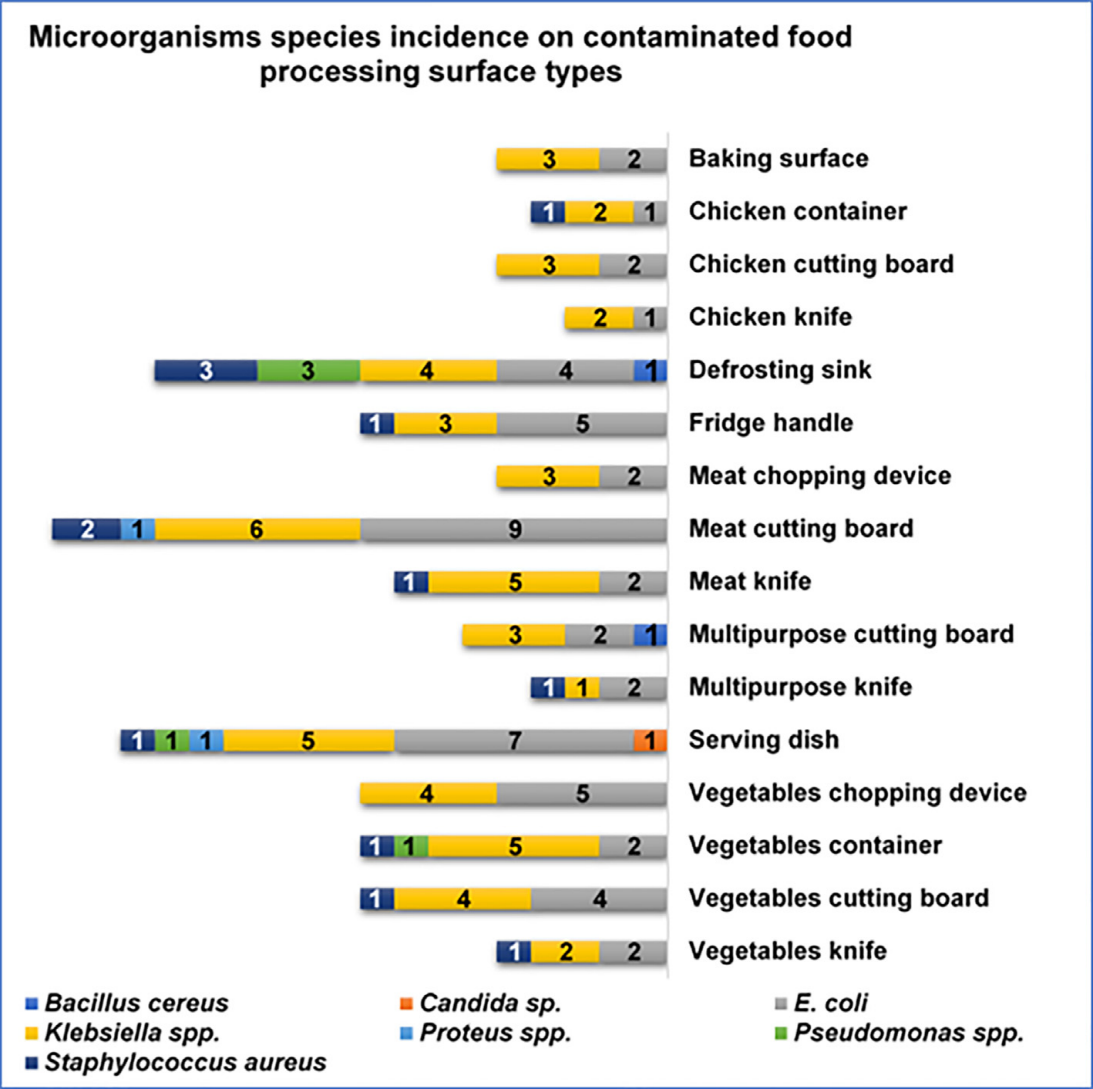
Incidence of contamination by each microorganism species on examined food processing surface types.

**Fig. 3:**
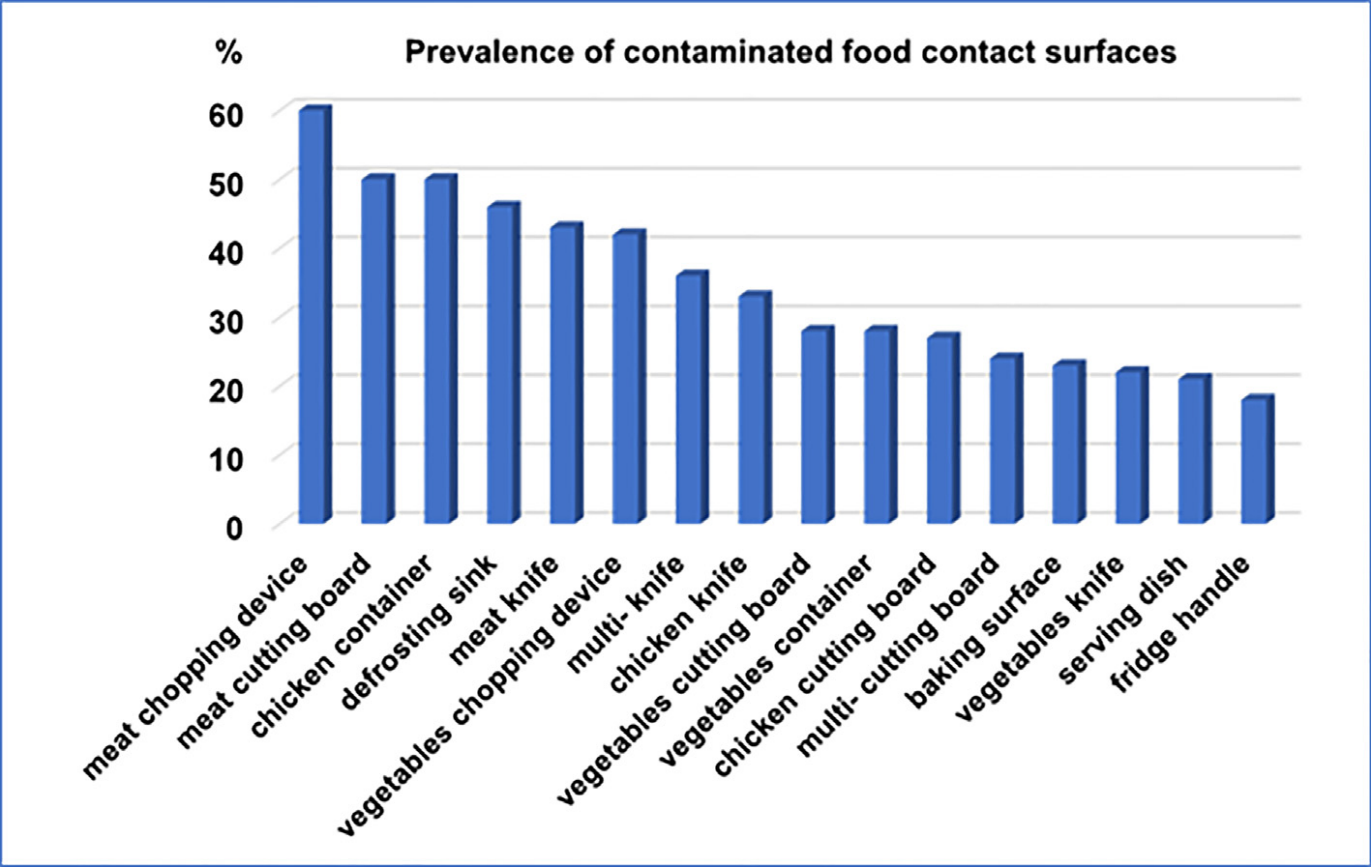
Prevalence of microbial contamination among each type of swabbed and examined food contact surface.

**Fig. 4:**
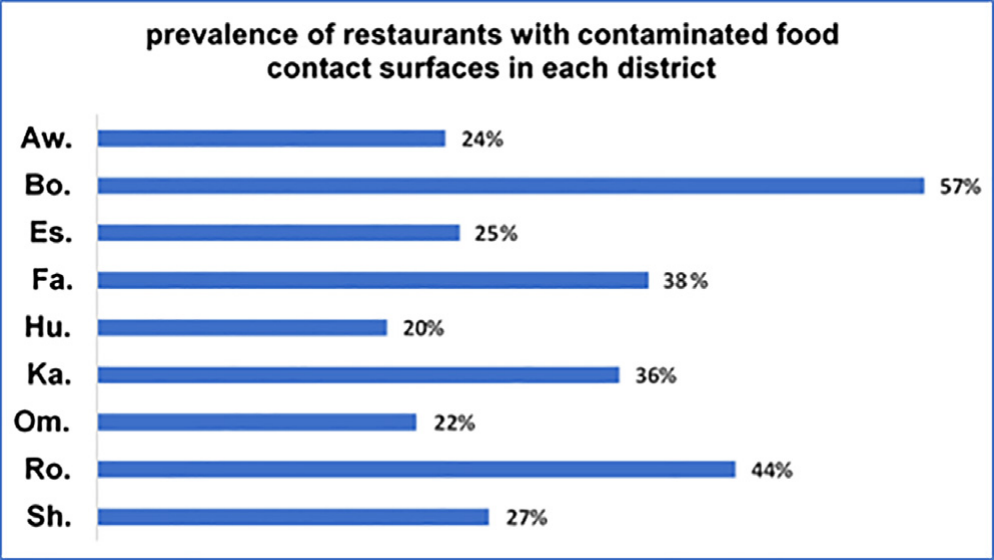
Prevalence of restaurants with at least one contaminated food contact surface by districts.

*Klebsiella* spp. and *Escherichia coli* were the most prevalent contaminating microorganisms, detected in 55/294 (18.7%) and 52/294 (17,7%) of sampled food contact surfaces, respectively. *Staphylococcus aureus* contamination was also relatively evident with an incidence of 4,4% (13/294). The analysis of the 294 collected samples, revealed the presence of *Pseudomonas* spp., *Proteus spp*., *Bacillus cereus*, and *Candida* sp. onto a small number of food contact surfaces: 5, 2, 2, and 1, respectively (Fig. 1). In this study, *E. coli* and *Klebsiella* spp. contaminant microorganisms were found in at least one of all sixteen types of investigated food contact surfaces (Fig. 2). No contamination with *Salmonella* spp. or *Listeria* spp. was detected. Meat chopping devices and cutting boards were found as the most contaminated food contact surfaces (60% and 50%), while washed serving dishes and fridge handles were the least contaminated (21% and 18%) (Fig. 3). The incidence of restaurants with contaminated food contact surfaces was significantly variable among the different districts, with a value as high as 57% in the most affected district and 20% in the less affected (Fig. 4).

## 4 Discussion

This study took place in Makkah city which considers one of the most crowded cities in the world. 294 samples from food preparation materials and storages were obtained to estimate the quality of the food industry. *Escherichia coli*, *Klebsiella* spp. and *Staphylococcus aureus* were found in 17,7%, 18.7% and 4,4% of sampled food contact surfaces, respectively. Many reported studies spotted significant levels of *E. coli* and *S. aureus* microorganisms, especially *E. coli* contaminations, in the food served by restaurants and catering establishments but also onto food processing surfaces and utensils (Mohamedin et al., 2015, Petruzzelli et al., 2018, Siti et al., 2018). Even though, *E. coli* and *Klebsiella* spp. were existed in at least one of all sixteen types of investigated food contact surfaces, highly food poisoning causing bacteria such as *Salmonella* spp. or *Listeria* spp. were spotted. However, the presence of fecal groups such as *E. coli* and *Klebsiella spp* in food or water indicates a likelihood of the occurrence of food poisoning causing bacteria such as *Salmonella* or *Shigella* as high as 96% when these contaminants are found in high counts (>1000 CFU/100 cm^3^) even though it decreases to about 54% when the number of detected fecal coliforms is smaller (<1000 CFU/100 cm^3^) (Cabral, 2010).

It has been largely approved that insufficient cleaning processes and bad storage conditions can result in such contamination with high microbial inhabitants especially on cutting boards (Little and Sagoo, 2009, RACGP., , 2014). In this case, the risk of foodborne illnesses could be escalated as studies documented that pathogenic bacteria such as *S. aureus, E. coli and Salmonella* spp. remain viable for a long time on food contact surfaces (Kusumaningrum et al., 2003). In this study, the high percentages of contamination in meat chopping devices and cutting boards can be attributed to previous reasons such as lack of hygiene and bad storage.

Most restaurants use primitive cleaning materials such as overused sponges and low-priced ineffective detergents, especially traditional neighborhood restaurants in olden districts where microbial contamination incidence was high in this study, in contrast to five stars' restaurants and branded fast-food restaurants chains who apply very strict quality control norms for cleaning and food processing and storage. It has been recommended that, where cleaning of utensils and surfaces is performed by traditional way such as washer’s sponge, hygiene level must be predominantly monitored to get satisfactory cleaning procedures (RACGP, 2014). However, the only solution to eradicate all contaminants from cooking surfaces and devices is by using recent methods such as alcohol spray and effective dishwashers (Rossvoll et al., 2015, Raghupathi et al., 2018). Furthermore, the training of food service workers and administrative improvement of the environment is very important to enhance the microbiological quality of food (Baluka et al., 2015, Al-Mazrous, 2004). Quality control, official inspection visits by authorities in charge, and requirement of accreditation certificates are effective remedies to improve sanitation level in food-serving establishments as was stated by many authors (Osimani et al., 2013, Garayoa et al., 2014).

Many precautionary procedures should be considered in food establishments to minimize contamination such as applying adequate physical and chemical barriers by using gloves, masks, alcohol spray, and glass shield, minding storage conditions by using separate refrigerators, and keeping storage areas away from food preparation ones; monitoring refrigerators temperatures, and periodic hand washing for involved staff (Todd et al., 2010).

## 5 Conclusions

The current study highlights the great disparities in the sanitation level of food contact surfaces among different restaurants in Makkah city. Although the overall findings indicated a low incidence of potential pathogens, it is highly recommended to increase the frequency of periodical microbiological assessment of sanitation levels, including onto food contact surfaces, in restaurants and catering establishments of the holy city. A strict application of an accreditation system such as the hazard analysis and critical control points (HACCP) plan would increase the cleanliness and sanitation level in the city restaurants.

## Declaration of Competing Interest

The authors declare the following financial interests/personal relationships which may be considered as potential competing interests: In behalf of all authors, I am the main author and correspondence Dr. Mamdouh Asaad Bukhari with Saudi national ID no. 1007348541 declare that there is no conflict of interest.
